# Goodness of fit to a mathematical model for Drosophila sleep behavior is reduced in hyposomnolent mutants

**DOI:** 10.7717/peerj.1533

**Published:** 2016-01-04

**Authors:** Joshua M. Diamond

**Affiliations:** Dornsife College of Letters, Arts and Sciences, University of Southern California, Los Angeles, CA, USA

**Keywords:** Sleep, Activity, Drosophila, Waking, Nonlinear regression, Architecture, Least-squares, *Insomniac*, Homeostasis

## Abstract

The conserved nature of sleep in Drosophila has allowed the fruit fly to emerge in the last decade as a powerful model organism in which to study sleep. Recent sleep studies in Drosophila have focused on the discovery and characterization of hyposomnolent mutants. One common feature of these animals is a change in sleep architecture: sleep bout count tends to be greater, and sleep bout length lower, in hyposomnolent mutants. I propose a mathematical model, produced by least-squares nonlinear regression to fit the form *Y* = *aX*^∧^*b*, which can explain sleep behavior in the healthy animal as well as previously-reported changes in total sleep and sleep architecture in hyposomnolent mutants. This model, fit to sleep data, yields coefficient of determination *R* squared, which describes goodness of fit. *R* squared is lower, as compared to control, in hyposomnolent mutants *insomniac* and *fumin*. My findings raise the possibility that low *R* squared is a feature of all hyposomnolent mutants, not just *insomniac* and *fumin*. If this were the case, *R* squared could emerge as a novel means by which sleep researchers might assess sleep dysfunction.

## Introduction

Sleep in Drosophila exhibits many characteristics that are seen also in mammalian sleep, including extended periods of quiescence and increased arousal threshold (Cirelli & Bushey, 2008). Following sleep deprivation, sleep in Drosophila is characterized by increased duration, hyper-consolidation, higher-than-usual arousal threshold, (Cirelli & Bushey, 2008) and decreased latency to sleep (Koh et al., 2008); all of these traits mirror mammalian response to sleep deprivation.

In addition to sharing behavioral characteristics, Drosophila and mammalian sleep appear to share similar underlying biochemical mechanisms, likely involving cAMP signaling. The adenylate cyclase mutant *rutabaga* demonstrates increased sleep, while the phosphodiesterase mutant *dunce* demonstrates reduced total sleep, suggesting a negative relationship between intracellular cAMP levels and sleep duration (Hendricks et al., 2001). Caffeine acts as a phosphodiesterase inhibitor (De Mendonça & Cunha, 2010), and thus might be expected to recapitulate the *dunce* phenotype. In fact, caffeine, which is a somnolytic agent in humans, also reduces Drosophila sleep length in a dose-dependent fashion (Hendricks et al., 2000). These findings suggest that increased cAMP activity may be sufficient to inhibit sleep in both Drosophila and in humans; moreover, they suggest a conserved biochemical mechanism for sleep in both species.

Behavioral and biochemical evidence for sleep as a conserved phenomenon has allowed Drosophila to emerge in the last decade as a powerful model for the study of sleep.

Much recent work in Drosophila has been focused on the study of hyposomnolent mutants (Koh et al., 2008; Hendricks et al., 2001; Stavropoulos & Young, 2011; Kume et al., 2005; Foltenyi, Greenspan & Newport, 2007). Study of sleep behavior in these mutants may shed light on the mechanisms of sleep and sleep pathology.

In addition to reduced total sleep, hyposomnolent mutants also demonstrate altered sleep architecture. Sleep is poorly consolidated: bout length is reduced as compared to control (Koh et al., 2008; Stavropoulos & Young, 2011; Foltenyi, Greenspan & Newport, 2007; Pfeiffenberger & Allada, 2012; Ueno et al., 2012). In some of these cases bout count is also reduced (Koh et al., 2008), but more frequently it is elevated (Stavropoulos & Young, 2011; Foltenyi, Greenspan & Newport, 2007; Pfeiffenberger & Allada, 2012). One such example is *insomniac* (Stavropoulos & Young, 2011; Pfeiffenberger & Allada, 2012), which is the basis of much of the modeling work in this study. *fumin*, which is also considered in this study, has been reported to demonstrate reduced sleep bout length (Ueno et al., 2012).

Several hyposomnolent mutants have also shown an absent or diminished sleep rebound following sleep deprivation (Koh et al., 2008; Kume et al., 2005; Foltenyi, Greenspan & Newport, 2007; Pfeiffenberger & Allada, 2012).

The goal of this study is to produce a mathematical model that describes the relationship between total sleep, sleep bout count and sleep bout length—during normal sleep and following sleep deprivation—in control animals. I will then examine the extent to which this model also holds true in hyposomnolent mutants. A mathematical model could provide better understanding of sleep behavior, in controls as well as in disease states. The extent to which the model holds true in mutant lines could be used as a measure of sleep function in those mutant lines.

Ueno et al. (2012) notes that, among individual Drosophila animals, the relationship between *sleep bout length* and *the probability of achieving a sleep bout of greater length* conforms to a power law function. The mathematical relationship between sleep bout length and *sleep bout count*, however, has not been considered in past work. Incorporating this relationship into my mathematical model is a goal of this study.

My results may establish a new paradigm for analysis of sleep dysfunction in hyposomnolent mutants. These techniques could also be used on higher animals, including humans.

## Methods

All animals came from the Bloomington Stock Center at Indiana University. *Insomniac* corresponds to stock number 18,307. w1118 was used as control.

*Insomniac* was outcrossed for 8 generations to an isogenic w1118 line to control for genetic background. Only males were used in this experiment, for mutants and controls. Animals were 1–5 days old.

Sleep was monitored using TriKinetics’ DAM2 Drosophila Activity Monitors, as previously described (Pfeiffenberger et al., 2010a). Briefly, animals were placed inside activity tubes containing food made of 5% sucrose and 2% agarose and then housed in an incubator with 12-hour:12-hour day:night cycles at 25 °C and 85% humidity. Sleep behavior was measured in parallel, in that both wildtype and *insomniac* were housed in the same incubator, at the same time, during the experiment. Animals were given three days to acclimate to the day/night cycle before data collection began. After the acclimation period, data collection lasted four full 24-hour periods. Sleep is defined as five minutes of inactivity (Pfeiffenberger et al., 2010b). Data was processed using SleepLab, custom Matlab-based software provided by Dr. William Joiner (UCSD). Animals that showed significant loss of health during the course of the experiment, as determined by the SleepLab software, were automatically excluded from the results.

Statistical analysis was handled with GraphPad PRISM 6. Daytime data have been separated from nighttime data, but otherwise all data have been combined together over the four days. In Ueno et al. (2012), the technique of combining data from multiple flies is used and is empirically validated.

Results are considered either as averages across the experiment or as individual animal data. An average across the experiment refers to the average of a specific measurement, taken over all animals of a specific genotype and over all days or nights of the experiment. For example, we might study mean total sleep of all *insomniac* animals during all nights of the experiment. We might also consider mean sleep bout length, which is more complicated. Animals tend to sleep multiple sleep bouts per day or night. Therefore, mean sleep bout length across the experiment is calculated by first averaging the length of all the sleep bouts slept by a given animal in a given day or night, and then by averaging again across all animals and all days or nights. Mean sleep bout length across the experiment thus refers to average of averages.

On the other hand, data analysis might consider individual animal data. Here, each data point is an animal-day or animal-night pairing. Each pairing consists of a total sleep, a *mean* sleep bout length (since, as mentioned earlier, sleep bouts tend to be multiple), and a sleep bout count.

This individual animal data is the basis for production of a mathematical model. Ordinary (unweighted) least-squares nonlinear regression is used to produce lines of fit, constrained to the equaiton *Y* = *aX*^∧^*b*. In each line of fit, the independent variable *X* represents the sleep bout count of the animal-time period pair, while the observed response variable *Y* represents the mean sleep bout length in that same animal-time period pair.

Similar lines of fit are produced using activity bout data. In activity bout data analysis, independent variable *X* represents activity bout count, and observed response variable *Y* represents mean activity bout length.

Similar lines of fit are also produced from sleep behavior the day following sleep deprivation. Wildtype and *insomniac* were observed in parallel for four days and three nights. On the fourth night, both genotypes were deprived of sleep throughout the 12-hour lights off period. Sleep deprivation was achieved by shaking the animals for two seconds every 20 s on average, with random sigma = 100%. Lines of fit are then produced so as to model sleep behavior during the fifth day, immediately following the night of sleep deprivation. Here, data is combined across multiple animals, but not across multiple days, since we only consider the single day immediately following the night of sleep deprivation. *n* = 7 control animals and 9 *insomniac* animals.

Finally, similar lines of fit are produced using data from Dr. William Joiner. Dr. Joiner contributed data from two experiments, both conducted in parallel, which compare the sleep behavior of *iso31*, a quasi-wildtype line, with that of *fumin*, a hyposomnolent mutant. *n* = 31 control animals and 28 *fumin* animals in the first experiment. *n* = 16 control and *fumin* animals in the second experiment.

Nonlinear regression assumes that the pool of residuals is drawn from a Gaussian distribution. The D’Agostino & Pearson omnibus K2 test is used to test for attainment of this requirement.

*R*^2^ is computed based on the data’s adherence to the same ordinary least-squares nonlinear regression line discussed premviously. Specifically, *R*^2^ = 1 − (SSres∕SStot), where SSreg is the sum of the squares of all distances along the *y*-axis between data points and the best-fit curve, and SStot equals the sum of squares of all distances along the *y*-axis between data points and the horizontal line that runs through the mean of all *y*-values.

## Results

*n* = 31 per genotype and 64 total. Since the experiment ran for four days and four nights, we consider sets of 31∗4 = 124 observations.

### Characterization of wild type sleep

**Figure 1:**
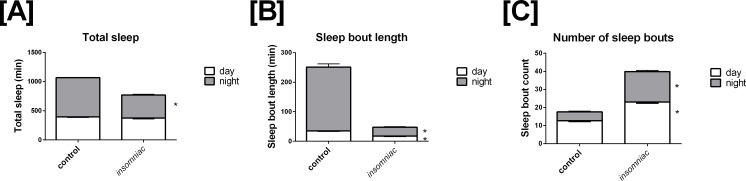
Characterization of sleep in *insomniac* versus control. Each genotype-time period pair represents an average across *n* = 124 measurements. (A) Total sleep in insomniac versus control. Values shown represent mean total sleep across the four days of the experiment. Nighttime total sleep is significantly decreased in *insomniac* as compared to control, while daytime sleep is unchanged. Also worthy of note is that total 24-hour sleep in *insomniac* is significantly decreased as compared control. This is not indicated in the figure. (B) Sleep bout length in insomniac versus control. Values represent averages across the length of the experiment. Sleep bout length is significantly reduced in *insomniac* as compared to control, for both the daytime and nighttime. (C) Mean length of sleep bouts across the length of the experiment. Sleep bout count is significantly increased in *insomniac* as compared to control, for both daytime and nighttime. ^∗^*p* < 0.0001 according to two-tailed, two-sample heteroscedastic Student’s *T*-test. Error bars represent the standard error measurement. Error bars for nighttime (gray) project above the top of the corresponding bar. Error bars for daytime (white) project from below the top of the corresponding bar.

Sleep in wild type animals is consistent with that seen in the literature, in terms of both total time slept and sleep architecture (Stavropoulos & Young, 2011; Pfeiffenberger & Allada, 2012). Total sleep in control is 396.4 min in the day, with SD = 82.9, and 672.6 min in the night, with SD = 29.9 (Fig. 1A). Total daily sleep is 1069.0 min, with SD = 81.7. Mean sleep bout length in control is 35.1 min in the day, with SD = 14.4, and 216.1 min in the night, with SD = 118.2 (Fig. 1B). Mean sleep bout count in control is 12.6 in the day, with SD = 4.6, and 4.9 in the night, with SD = 4.2 (Fig. 1C).

### Characterization of *insomniac* sleep

*Insomniac* demonstrates a robust phenotype in terms of total time slept. *Insomniac* animals tested in this experiment sleep significantly less than controls in the 24-hour period. Mean total sleep in *insomniac* is 770.1, with SD = 210.0, compared to 1069.0 with SD = 81.7 in wild type (Fig. 1A). According to a two-tailed, two-sample heteroscedastic (allowing for unequal variance) Student’s *T*-test, probability that measures of total sleep per 24 h in *insomniac* and controls came from the same distribution is given by *p* < 0.0001. Separate consideration of daytime and nighttime sleep reveals that nighttime total sleep in *insomniac*, at 394.7 (SD = 148.7) is significantly less than nighttime sleep in wildtype, at 672.6 (SD = 29.9) (Fig. 1A). Daytime total sleep in *insomniac* is unchanged as compared to control (Fig. 1A).

*Insomniac* also demonstrates a strong phenotype in sleep architecture. Bout length is shorter and bout count greater in *insomniac* as compared to its control. Mean sleep bout length in *insomniac* is 17.4 in the day, with SD = 8.4, and 30.0 in the night, with SD = 21.4 (Fig. 1B). Both of these values are significantly reduced as compared to wild type. Meanwhile, bout count is significantly greater in *insomniac*, with mean bout count = 23.1 (SD = 8.1) in the day and 16.8 (SD = 7.5) in the night (Fig. 1C).

**Figure 2:**
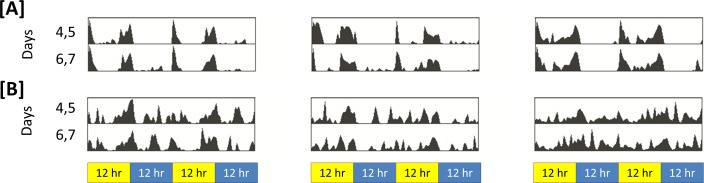
Representative actograms for control and *insomniac*. (A) control. (B) insomniac. Each panel represents the sleep/wake activity of a single animal. So, three animals are shown for each genotype, and six are represented in total. Note disorganized sleep/wake behavior in *insomniac*, including extensive activity during lights-off 12-hour periods.

That sleep in *insomniac* is poorly consolidated can be observed qualitatively. Figure 2 represents activity in *insomniac* and control. We see that, in the case of *insomniac*, activity is distributed throughout periods in which control flies normally sleep.

### Production of a mathematical model

**Figure 3:**
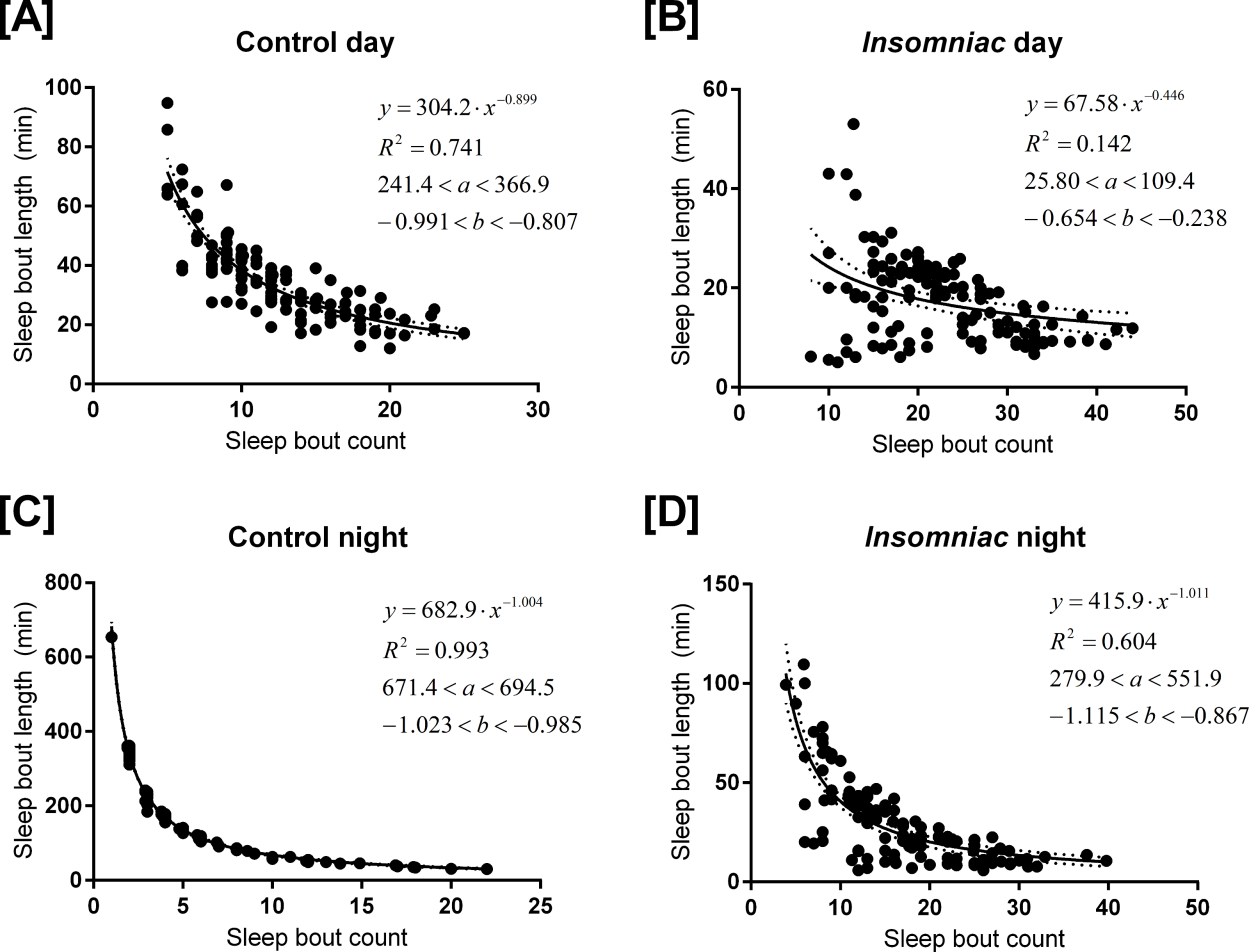
Relationship between sleep bout count and mean sleep bout length in individual control and *insomniac* animals. Each dot represents a single animal-day pair (A and B) or animal-night pair (C and D). The *y* axis represents the mean length of sleep bouts achieved during each animal-time period pair, and the *x* axis represents the amount of sleep bouts achieved in that same animal-time period pair. Thus *n* for each figure is equal to 31∗4 = 124 animal-time period pairs. Each panel contains an inset, which lists, from to bottom: the equation of the line of fit, in the format *Y* = *aX*^∧^*b*; the coefficient of determination *R*^2^; the 95% confidence interval for the *a* parameter; and the 95% confidence interval for the *b* parameter. Dotted lines represent the upper and lower margins of the 95% confidence band. The chances are 95% that the true line of fit lies between these upper and lower margins.

Figure 3 shows sleep bout count and sleep bout length for each animal-day or animal-night pair in either *insomniac* or control lines.

Sleep behavior is most regular in the case of control night (Fig. 3C). To this set of data, I fit the model

(1) \begin{eqnarray*} y=a\cdot {x}^{b} \end{eqnarray*}

where *y* corresponds to mean sleep bout length, for an individual animal, over the course of a single night; and *x* corresponds to sleep bout count for that same individual animal over the course of a single night.

I use the coefficient of determination *R*^2^ to assess the strength of this model. *R*^2^, in short, describes the vertical distance from the data points to the line that attempts to approximate them. If the line provides a good approximate, the data points will tend to be close to the line, and so this vertical distance will tend to be small. A high *R*^2^ indicates a low vertical distance, on the whole, and thus it indicates that the line of fit approximates the data well.

The coefficient of determination *R*^2^ is 0.993 in the case of control night, indicating that Eq. (1) provides a good approximate to these data. In Fig. 3, Eq. (1) is fit to all experimental conditions. *R*^2^ is not as high in other experimental conditions as it is in control night (Fig. 3C), indicating a worse fit to the model in these other experimental conditions.

Equation (1), the parameters that comprise it, and the *R*^2^ coefficient might provide valuable insight towards the analysis of sleep behavior in Drosophila, even in experimental conditions where *R*^2^ is relatively low.

The parameter *b* is negative in experimental conditions. This indicates that, as bout count rises, mean sleep bout length falls. Further, *b* tends to reside near −1.

In Eq. (1), *a* tends to estimate total sleep. For example, in Fig. 3C, *a* = 682.9. Consistent with this prediction, measured sleep for this genotype and timeframe is 672.6 min. Given the form of Eq. (1), one ought to expect that the parameter *a* would estimate total sleep. Suppose, in the regression Eq. (1), it so happens that *b* = − 1 exactly. Then we can re-express the equation as

(2) \begin{eqnarray*} y\cdot x=a. \end{eqnarray*}

Equation (2) shows that (in the case *b* = − 1) the best regression generates a fixed constant *a* with the special property that the product of any pair of values attained by the variables *x* and *y* tends to fall close to *a*. These values in turn correspond to the bout count and mean bout lengths, respectively, of the animals. And, we know that, in an individual animal-time period pair, mean bout length times bout count equals total sleep for that time period. Thus we see why, when *b* falls close to −1, *a* estimates total sleep.

As *b* deviates from −1, *a* becomes a worse estimate of mean total sleep. For example, in Fig. 3B, *a* = 68. This drastically underestimates total sleep for this genotype and timeframe. For *b* > − 1, *a* is an underestimate of mean total sleep. For *b* < − 1, *a* is an overestimate. The tendency of *a* to estimate total sleep, as well as the relationship between *b* and *a* I have just described, holds in both control animals and in *insomniac*. In *insomniac*, *a* may not be as good an estimate of total sleep, in part because *b* may stray further from −1.

The coefficient of determination *R*^2^ may be of use. As described earlier, *R*^2^ is greatest in the setting of control sleep behavior at night. *R*^2^ close to 1 indicates that the mathematical model closely fits the data.

*R*^2^ is closer to 1 in the nighttime, as compared to the daytime, with genotype controlled for. In other words, control night has greater *R*^2^ than control day; meanwhile, *insomniac* night has greater *R*^2^ than *insomniac* day. Additionally, *R*^2^ is farther from 1 in *insomniac*, as compared to control, with time of day controlled for. *Insomniac* night has lower *R*^2^ than control night; *insomniac* day has lower *R*^2^ than control day.

So, in the daytime, and in *insomniac*, the model tends to fit the data less well.

Under conditions where *R*^2^ is relatively low, such as *insomniac day*, 95% confidence intervals for parameters *a* and *b* tend to be wider relative to the absolute value of these parameters. Also, 95% confidence bands tend to be wider as well in conditions with low *R*^2^.

### Statistical tests for the appropriateness of the mathematical model

Dependency between parameters *a* and *b* as they fit to the sleep data ranges from 0.822 to 0.984.

The sleep data do not pass the D’Agostino & Pearson omnibus K2 test of normalcy test in any genotype or timeframe, including control day, control night, *insomniac* day, and *insomniac* night.

### Application of the model to activity data

I conducted a similar statistical analysis on the behavior of the animals used in this experiment, except considering activity bouts as opposed to sleep bouts.

Equation (1) does not fit the activity bout data as well as it fits the sleep bout data. *R*^2^ is 0.608 at maximum.

Like in the case of the sleep bout data, *R*^2^ is higher in control than it is in *insomniac*. *R*^2^ values are 0.635 and 0.637 in control, daytime and nighttime, respectively (Figs. S1A and S1C), compared to 0.408 and 0.325 in *insomniac* (Figs. S1B and S1D).

Note that, in contrast with the sleep bout data, it is not the case in the activity bout data that *R*^2^ changes in daytime as compared to nighttime. Within a given genotype, daytime and nighttime *R*^2^ values are nearly identical.

### Application of the model to sleep rebound

**Figure 4:**
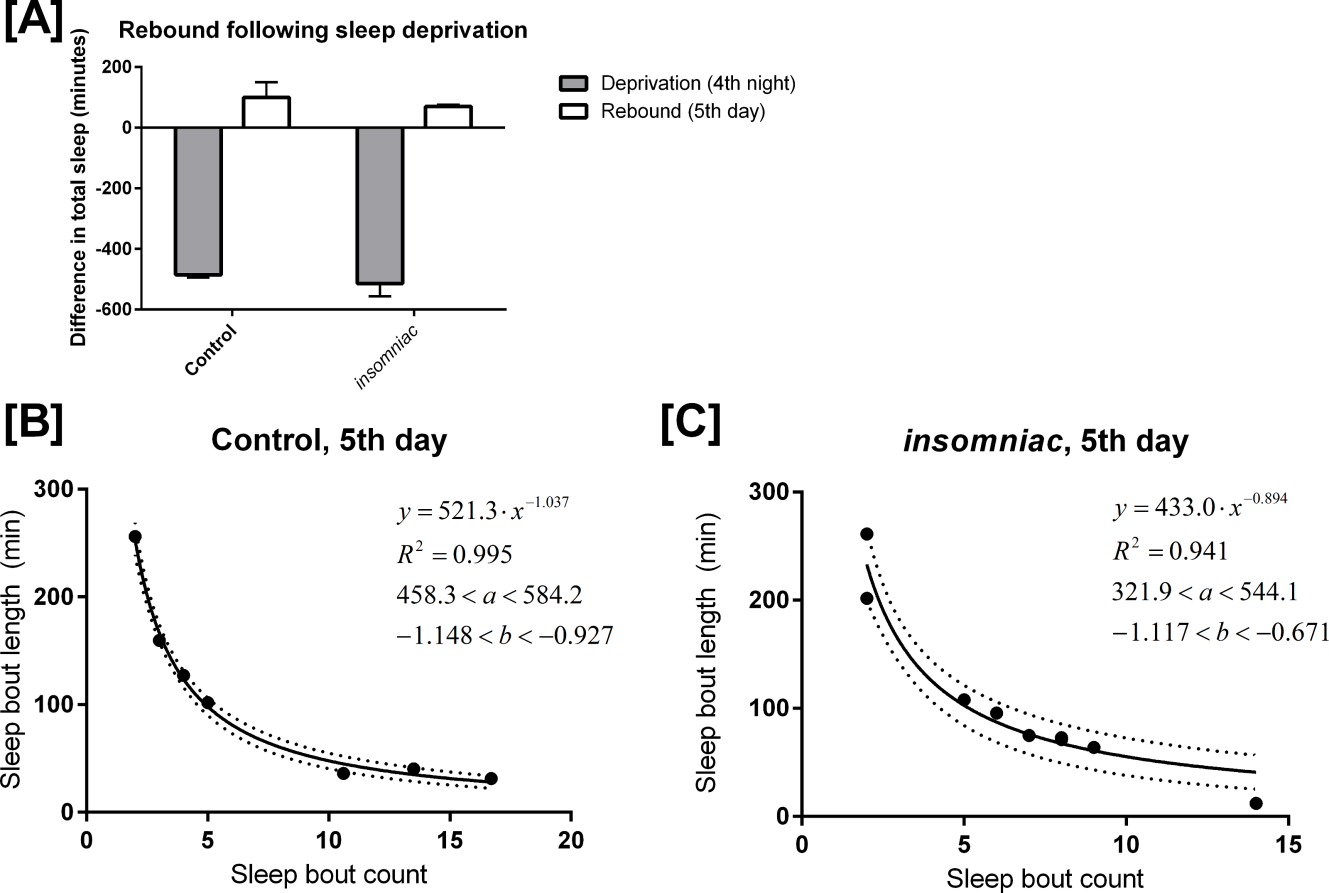
Mathematical model applied to sleep rebound following deprivation. (A) shows sleep deprivation and subsequent rebound in control and *insomniac*. The gray bar represents the difference in mean total sleep during the fourth night as compared to the mean total sleep in the first three nights. The white bar represents mean total sleep during the fifth day as compared to the mean total sleep during the first three days. Error bars represent SEM. (B) and (C) show the mathematical model as applied to sleep behavior during this fifth day. Each dot represents a single animal-day pair. The *y* axis represents the mean length of sleep bouts achieved during each animal-day pair, and the *x* axis represents the amount of sleep bouts achieved in that same animal-day pair. *n* = 7 animal-day pairs in (B) and 9 animal-day pairs in (C). Each panel contains an inset, which lists, from to bottom: the equation of the line of fit, in the format *Y* = *aX*^∧^*b*; the coefficient of determination *R*^2^; the 95% confidence interval for the *a* parameter; and the 95% confidence interval for the *b* parameter. Dotted lines represent the upper and lower margins of the 95% confidence band.

Control and *insomniac* animals were deprived of sleep throughout the entire 12-hour lights off period on the fourth night of an experiment. On the fifth day, both control and *insomniac* animals demonstrate sleep rebound, but *insomniac* sleep rebound is diminished as compared to wildtype (Fig. 4A). On night four of the sleep deprivation experiment, control slept 9.1 min on average (SD = 21.6), which is 486 min fewer than the mean sleep time for the first three nights of the experiment. Meanwhile, on night four, *insomniac* slept 3.4 min on average, which is 514 min fewer than the mean sleep time for the first three nights of the experiment. Then, on day five, following deprivation, control slept 496 min on average, with SD = 50.2. This is 100 min greater than the mean sleep time in control for the first three days of the experiment. *insomniac*, on day five, slept 496 min on average, with SD = 126. This is 70.6 min greater than the mean sleep time in *insomniac* for the first three days of the experiment.

I tested my model on the daytime sleep behavior during this fifth day, immediately following sleep deprivation. The results are shown in Figs. 4B and 4C. Equation (1) predicts sleep behavior well during sleep rebound. In fact, *R*^2^ is *higher* following rebound than it is during usual daytime sleep for each respective genotype. Compare Fig. 4B with Figs. 3A and 4C with Fig. 3B.

The finding discussed in ‘Production of a mathematical model’, where *R*^2^ is lower in *insomniac* than it is in control, still holds in the case of sleep rebound.

### Application of the model to other lines

Trikinetics data from two other lines was obtained from Dr. William Joiner. These are *iso31*, a quasi-wildtype line which serves as control, and *fumin*, a hyposomnolent line. Two experiments, both conducted in parallel, compare sleep behavior in *iso31* with sleep behavior in *fumin*.

Sleep time in control 500.1 min in the day with SD = 56.1, and 559.5 min at night with SD = 49.5 in the first experiment. In the second, sleep time in control is 508.4 min in the day with SD = 46.5, and 561.6 min at night with SD = 49.6. Sleep time is drastically reduced in *fumin*, more than it is in *insomniac*. In the first experiment, sleep is 199.4 min in the day (SD = 80.3) and 142.1 min at night (SD = 115.4). In the second, sleep is 198.7 min in the day (SD = 90.24) and 92.7 min in the night (SD = 99.1).

Regarding *R*^2^, similar results to those described in ‘Production of a mathematical model’ are also found in Dr. Joiner’s data.

*R*^2^ for *iso31* is very high, with *R*^2^ = 0.890 in the daytime and *R*^2^ = 0.980 in the nighttime, in the first experiment (Figs. S2A and S2C). In the second experiment, *R*^2^ is 0.921 and 0.960 in daytime and nighttime respectively. *R*^2^ in *fumin*, on the other hand, is lower even than the *R*^2^ values observed in *insomniac*. In the first experiment, *R*^2^ = 0.009 and 0.058 in the daytime and nighttime, respectively (Figs. S2B and S2D). In the second, *R*^2^ = 0.022 and 0.006 in the daytime and nighttime, respectively.

As in ‘Production of a mathematical model,’ *R*^2^ tends to be greater for nighttime sleep than for daytime sleep. This is true in the first experiment universally, and in the second experiment in control but not in *fumin*.

## Discussion

### Evaluation of sleep behavior

Sleep behavior in control is normal quantitatively (Fig. 1) and qualitatively (Fig. 2). This indicates that my sleep system is in good working order. Further, the sleep phenotype I have demonstrated in *insomniac* mutants, which is characterized by reduced total sleep and poor consolidation, is consistent with past reports (Stavropoulos & Young, 2011; Pfeiffenberger & Allada, 2012).

### Merits of the mathematical model

Coefficient of determination *R*^2^, which measures goodness of fit to the mathematical model described in Eq. (1), is as high as 0.993. This serves to validate the mathematical model: at least in some circumstances, the model describes behavior very well. Even in conditions where *R*^2^ is not as high, such as in *insomniac* night or control day, the model appears to describe the behavior reasonably well considering the higher degree of variability within those data.

Note that *R*^2^ constitutes a measure of sleep behavior independent of those measures usually studied in Drosophila sleep research, namely, total sleep, mean bout length, and mean bout count. Any of these measures could be changed in a Drosophila line, without change in *R*^2^. Likewise, *R*^2^ could theoretically change without corresponding change in total sleep, mean bout length, or mean bout count. Thus, the *R*^2^ measure offers novelty.

As an alternative to *R*^2^, the model also yields 95% confidence intervals for parameters *a* and *b*. As mentioned in the results, where *R*^2^ is relatively low, the 95% confidence intervals for parameters *a* and *b* tend to be wide relative to the absolute value of these parameters. So, Eq. (1) parameter confidence interval width could also serve as a novel measure of sleep dysregulation. Confidence bands also tend to be wider in situations with low *R*^2^.

### Limitations of the model

Dependency between parameters *a* and *b*, when used to describe sleep behavior in *insomniac* and control animals, can be as high as 0.984. This indicates that *a* and *b* may be redundant. If a simpler model is desired, Eq. (2) would suffice. However, the inclusion of *b* seems to be merited, because production of a model conforming to Eq. (1) is not difficult, and *b* still improves goodness of fit.

That the sleep data universally fail the D’Agostino & Pearson omnibus K2 test under all circumstances might be cause for concern. Regardless of this finding, though, my model still appears to have merit, discussed in ‘Merits of the mathematical model’. Further, failure of this test need not indicate that nonlinear regression is an inappropriate strategy. Especially in large data sets, deviations from normalcy may reach statistical significance without corresponding to real practical meaning (D’Agostino, 1986). Therefore, it appears that my least-squares nonlinear regression procedure may be resistant to violations of the standard that underlying distributions be Gaussian (D’Agostino, 1986). Nevertheless, future work could look at the use of robust nonlinear regression models, as opposed to the least-squares nonlinear model used here. These are less distorted by data sets whose residuals come from non-Gaussian distributions (D’Agostino, 1986).

Also note that, if mean sleep bout length values are weighted by 1∕*y*^2^, performance on the D’Agostino & Pearson omnibus K2 normalcy test is improved but still poor.

### Teleological significance of the model

That Eq. (1) provides a good fit to the data—at least under some circumstances, like control night (Fig. 3C)—indicates that bout length and bout count are regulated with respect to each other such that, despite substantial variability in the values of these measures, total sleep tends to fall within a narrow range. Recall Eq. (2): if Eq. (2) provides a good fit to a set of animals, then no matter how much bout length and bout count vary among those animals, that each individual’s total sleep will be close to *a*.

So, *R*^2^ may be indicative of how tightly bout length and bout count are regulated among a group of individuals so as to produce levels of total sleep within a narrow range. High *R*^2^ could serve as a marker for successful regulation of sleep. A low *R*^2^ would suggest decreased regulation of sleep or an interference with the ability to regulate sleep.

*R*^2^ for daytime sleep is less than *R*^2^ for nighttime sleep (‘Production of a mathematical model’). This might then suggest that daytime sleep is less tightly regulated than nighttime sleep.

*R*^2^ for activity data is less than *R*^2^ for sleep data (‘Application of the model to activity data’). This could suggest that time spent active is less tightly regulated than time spent asleep.

*R*^2^ for sleep rebound is greater than *R*^2^ for normal daytime sleep (‘Application of the model to sleep rebound’). This could suggest that sleep after a period of sleep deprivation is more strictly regulated than is sleep without sleep deprivation.

Finally, *R*^2^ is low in *insomniac* (‘Production of a mathematical model’) and in *fumin* (‘Application of the model to other lines’), and it is lower in the latter than in the former. Meanwhile, mean total sleep is also lower in *fumin* than in *insomniac*. This suggests a sort of *dose-dependent* relationship between sleep impairment and *R*^2^, where, as total 24-hour sleep falls, so does *R*^2^. The more total sleep is impaired, the more achievement of tightly-regulated sleep also tends to be impaired.

Overall, these results suggest that *R*^2^ could serve as a measure for the extent of sleep regulation. This is true in Drosophila as well as in higher animals.

## Supplemental Information

## Additional Information and Declarations

### Data Availability

The following information was supplied regarding data availability: Raw data can be found in the Supplemental Information.
