# Supplementary figures and images for: Goodness of fit to a mathematical model for Drosophila sleep behavior is reduced in hyposomnolent mutants
Source: PeerJ. 2016 Jan 4;4:e1533. doi: 10.7717/peerj.1533 (PMC4800425; doi:10.7717/peerj.1533)

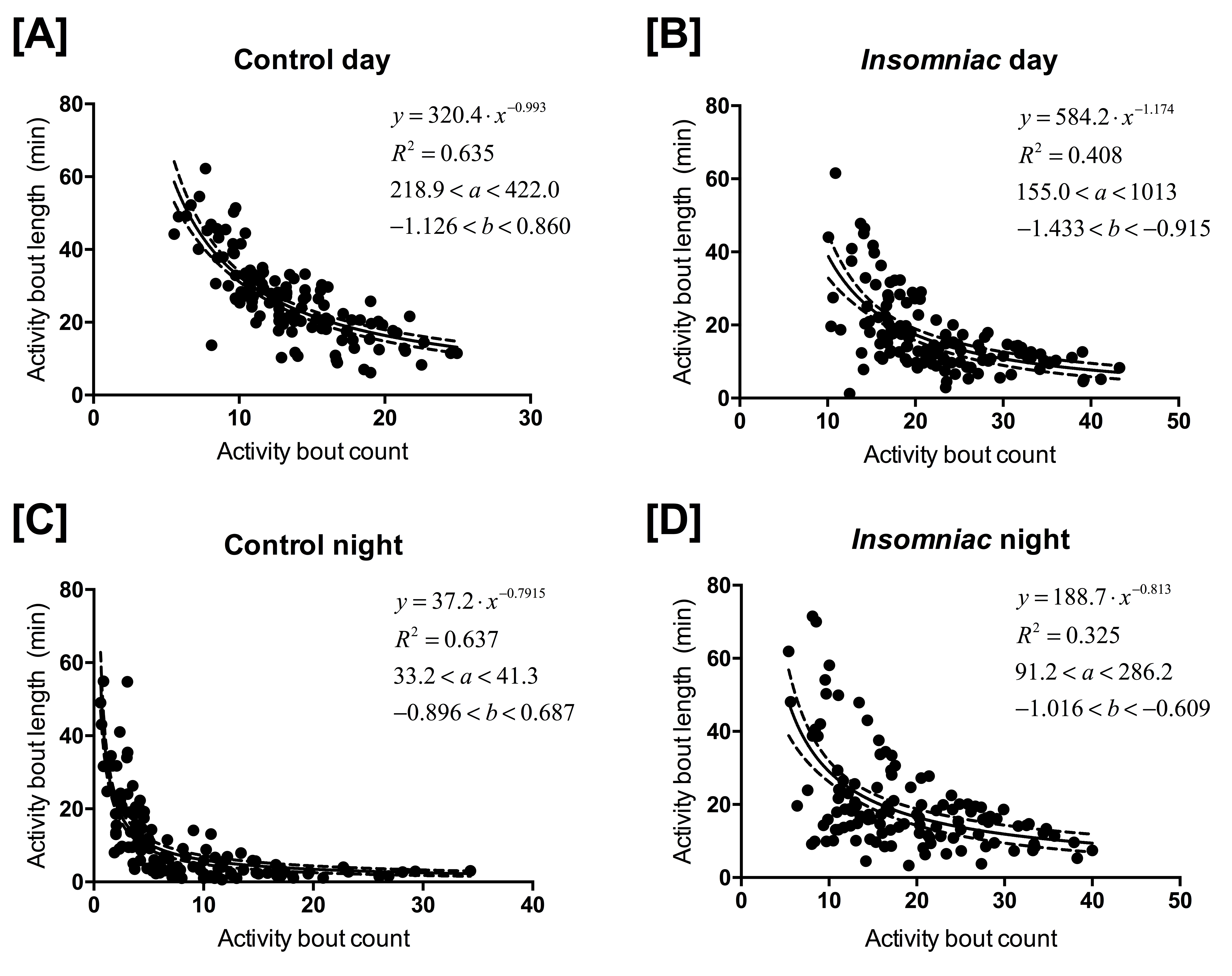

Supplement: Supplemental Information 1 — Each dot represents a single animal-day pair (A and B) or animal-night pair (C and D). The y axis represents the mean length of activity bouts achieved during each animal-time period pair, and the x axis represents the amount of activity bouts achieved in that same animal-time period pair. Thus n for each figure is equal to 31∗4 = 124 animal-time period pairs. Each panel contains an inset, which lists, from to bottom: the equation of the line of fit, in the format Y = aX∧b; the coefficient of determination R2; the 95% confidence interval for the a parameter; and the 95% confidence interval for the b parameter. Dotted lines represent the upper and lower margins of the 95% confidence band. The chances are 95% that the true line of fit lies between these upper and lower margins. [file peerj-04-1533-s001.png]

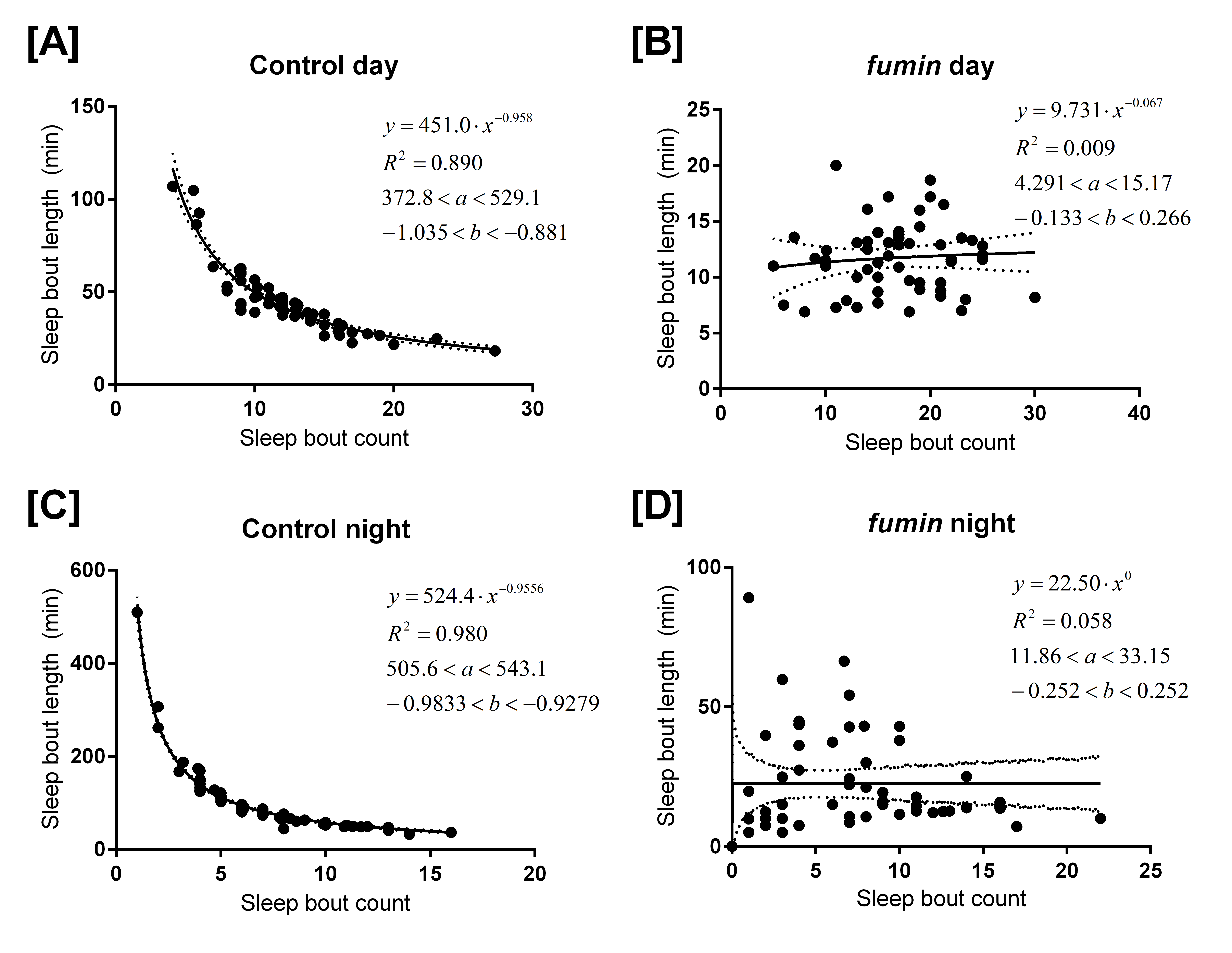

Supplement: Supplemental Information 2 — Each dot represents a single animal-day pair (A and B) or animal-night pair (C and D). The y axis represents the mean length of sleep bouts achieved during each animal-time period pair, and the x axis represents the amount of sleep bouts achieved in that same animal-time period pair. n for control (A and C) is 31∗4 = 124 animal-time period pairs. n for fumin (B and D) is 28∗4 = 112 animal-time period pairs. Each panel contains an inset, which lists, from to bottom: the equation of the line of fit, in the format Y = aX∧b; the coefficient of determination R2; the 95% confidence interval for the a parameter; and the 95% confidence interval for the b parameter. Dotted lines represent the upper and lower margins of the 95% confidence band. [file peerj-04-1533-s002.png]
